# Improving the stability of plasmonic magnesium nanoparticles in aqueous media†

**DOI:** 10.1039/d1nr06139a

**Published:** 2021-11-29

**Authors:** Jérémie Asselin, Elizabeth R. Hopper, Emilie Ringe

**Affiliations:** Department of Materials Science and Metallurgy, University of Cambridge Cambridge CB3 0FS UK er407@cam.ac.uk +44 (0)1223334567 +44 (0)1223334300; Department of Earth Sciences, University of Cambridge Downing Street Cambridge CB2 3EQ UK; Department of Chemical Engineering and Biotechnology, University of Cambridge Cambridge CB3 0AS UK

## Abstract

This work describes two different core–shell architectures based on Mg nanoparticles (NPs) synthesised in order to improve Mg's stability in aqueous solutions. The shell thickness in Mg–polydopamine NPs can be modulated from 5 to >50 nm by ending the polymerization at different times; the resulting structures stabilize the metallic, plasmonic core in water for well over an hour. Mg–silica NPs with shells ranging from 5 to 30 nm can also be prepared *via* a modified Stöber procedure and they retain optical properties in 5% water-in-isopropanol solutions. These new architectures allow Mg nanoplasmonics to be investigated as an alternative to Ag and Au in a broader range of experimental conditions for a rich variety of applications.

## Abstract 2

Plasmonic Mg nanoparticles can be stabilised up to a few hours in aqueous suspensions by protecting them inside core–shell architectures, which are prepared by condensation of either polydopamine or sol–gel silica.

## Graphical abstract

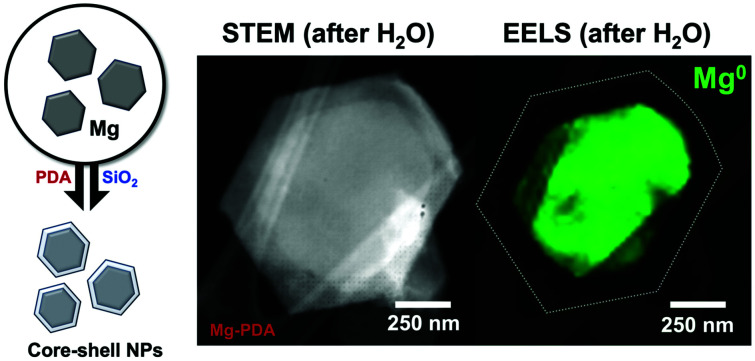

## Introduction

Magnesium is a new alternative plasmonic material with exceptional potential for harnessing energy across the broad UV-visible-NIR range, as signaled by its good plasmonic quality factor, defined as the ratio of the real and the imaginary parts of a material's dielectric function.^1^ Its inexpensive synthesis at large scale and sustainable future availability in comparison to noble metal compositions add to Mg's attractiveness.^2^ Further, its hexagonal close-packed (HCP) crystal structure, distinct from that of the other plasmonic metals, enables the formation of different and new nanoparticle (NP) shapes ranging from hexagonal nanoplates, nanorods, and novel folded structures called tents, chairs, tacos, and kites.^3–5^

Mg is well known as a rather reactive element. Its initial uses in the nanoparticulate form, mostly fabricated with top-down approaches, have been for hydrogen sensing and storage, where the non-plasmonic hydride (MgH_2_) can reversibly release hydrogen and regenerate metallic Mg in the presence of a catalyst.^6^ The electrochemical reactivity of colloidal Mg – using its strong reducing potential as driving force – leads to rapid galvanic replacement with other metals, such that Mg can be used either as a sacrificial template^7^ or a scaffold for partial replacement leading to multifunctional, multimetallic architectures.^8^

While Mg's reactivity can be utilised for syntheses, it renders Mg^0^ intrinsically unstable in a number of common conditions. Indeed, metallic Mg tends to oxidize spontaneously to form a thin layer of MgO when in contact with air and Mg(OH)_2_ when in contact with water. Fortunately, this 10 nm thick oxide acts as a passivating, self-limiting layer, allowing Mg NPs to remain metallic and plasmonic in a range of anhydrous conditions.^3^ However, the instability of Mg and MgO towards water, leading to the formation of a soluble hydroxide, hinders the dispersion of as-synthesised Mg NPs in aqueous conditions.

Core–shell architectures aiming to protect and functionalize have emerged for reactive metals, including plasmonic nanomaterials; the shell composition can be adapted to the desired application and dispersion conditions.^9–13^ Shells allow for expanded applications of plasmonic materials, with possibilities for improved biocompatibility, biosensing *via* fluorescence^14–17^ or Raman scattering,^15,18^ targeted drug delivery, and enhanced colloidal and chemical stability, to name a few.^19–22^ Common shell compositions rely on the condensation of inorganic oxides,^9,15,16,23,24^ including silica (SiO_2_), and polymeric compounds.^25^ The Stöber methodology for silica coating has been broadly used for different nanomaterials to produce shell-like or colloidal silica with controlled thickness/size, porosity, and electrostatic charge.^23,26^ The chemistry of silanes being well developed, such shells allow for a wide range of chemical functionalization. An alternative is the synthesis of organic polymer shells from their monomers. Shell formation relies on controlled *in situ* polymerization that commonly requires priming of the NP surfaces prior to a reaction with an available monomeric species. Polymer shells can be convenient for applications where shell hydration or swelling is relevant or useful, as well as in biological sensing, targeting, and drug delivery.^27–29^ However, this multi-step polymerization strategy leads to porous layers that are not suitable for the protection of an easily oxidizable core like Mg. In recent years, self-polymerization of dopamine initiated by a change in pH has been used to produce a cross-linked layer with increased stability. This polydopamine (PDA) coating improves the biocompatibility of core–shell architectures and promotes reactivity with amine-rich biomolecules such as antibody complexes, aptamers, and oligomers.^30–33^ Moreover, both reduced and oxidized PDA have fluorescent properties with excitation in the ultraviolet and emission in the visible wavelength range.^34^

Here, we investigate the Mg NP aqueous stability improvement bestowed by two common shell compositions – polydopamine and silica (SiO_2_). We study the parameters affecting both core–shell syntheses, and choose a single condensation step for the shell in order to minimize the oxidation of Mg cores during the process. We find that the final thickness can be tuned by controlling either the reaction duration (for PDA) or the precursor concentration (for SiO_2_), and that PDA is effective as a short to medium-term barrier to oxidation. This work enables a variety of sensing and biological applications by stabilizing NPs in water for sufficient time for their use, after which their core would safely degrade as biocompatible cations.

## Results and discussion

### Magnesium@polydopamine core–shell particles

**Fig. 1:**
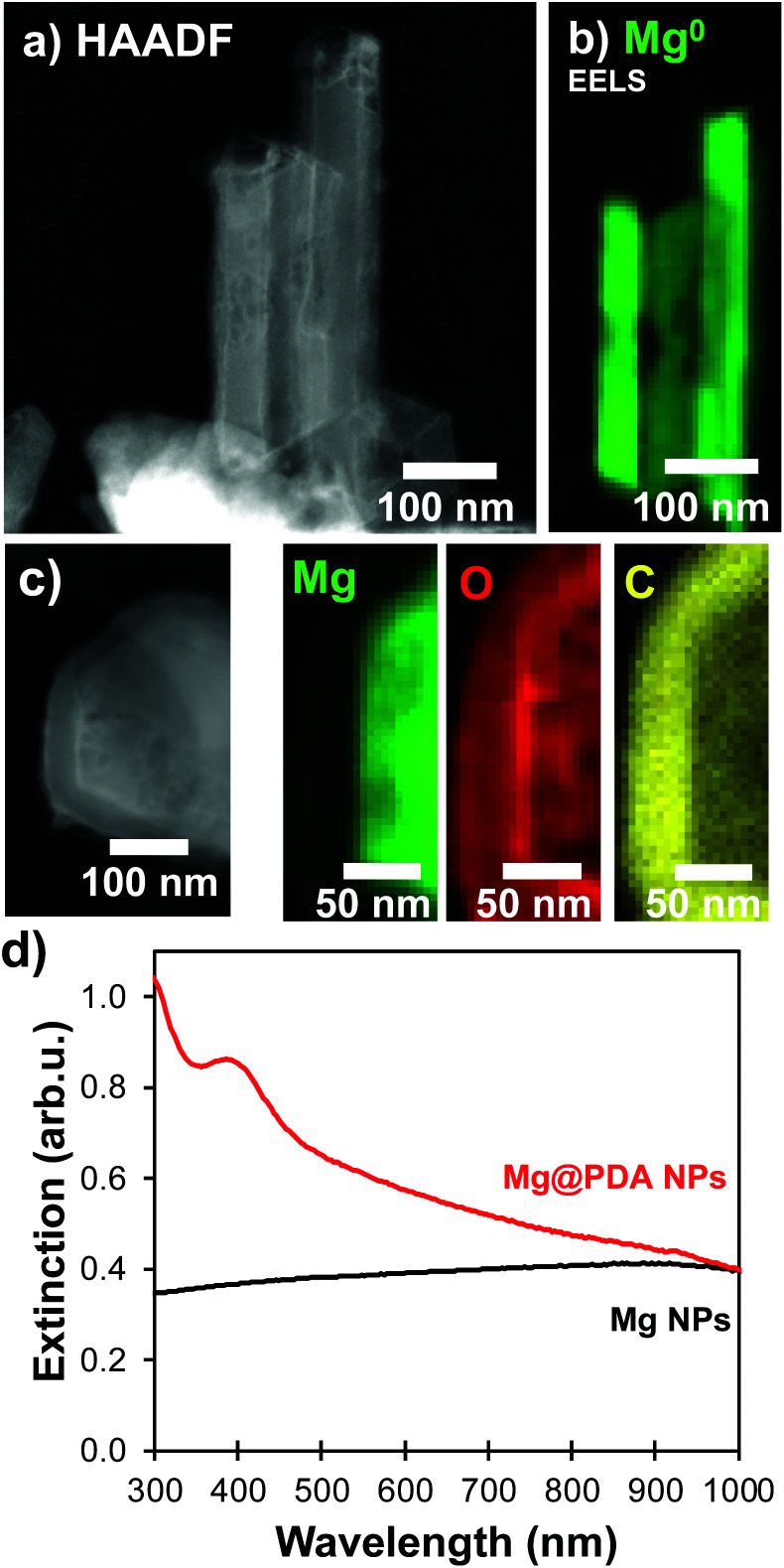
PDA shells on Mg NPs. (a) STEM-HAADF, (b) STEM-EELS signature of the Mg bulk plasmon (10.6 eV), (c) STEM-EDS maps for K_α_ lines of Mg (1.25 keV), O (0.52 keV), and C (0.28 keV), and (d) extinction signature in UV-VIS spectrometry before (black) and after (red) the dopamine polymerization.

The catechol-directed polymerization of dopamine hydrochloride in alkaline conditions has been investigated extensively and has found applications in the coating of nanomaterials for improved biocompatibility, drug-delivery, stability, sensing, and fluorescence.^25,31–33^ Since Mg NPs are poorly stable in the pH 8.5 trisaminomethane-buffered aqueous medium usually needed for dopamine polymerization, we adapted the reaction to water-free organic conditions. A weak base can instead be introduced in ethanol to initiate the polymerization on suspended NPs. Using a 4 mg mL^−1^ concentration of dimethylamine (DMA) as a base catalyst in ethanol leads to coatings and reaction times similar to that of Au@PDA NPs from literature.^32^ No secondary nucleation of PDA NPs was observed in scanning transmission electron microscopy (STEM) and a uniform shell was prepared over the Mg NP cores (Fig. 1a). These samples were composed of a mixture of single and aggregated NPs, both of which showed similar shell thicknesses around and between the Mg cores.

The addition of a PDA shell did not disrupt the metallic character of the Mg core: electron energy loss spectroscopy (EELS) can be used to probe the oxidation level of elements *via* their electronic absorption signatures. For instance, metals such as Mg^0^ and Al^0^ display prominent bulk plasmons in the 10–20 eV region signaling their metallic character.^35,36^ In a given metal, bulk modes are distinct in energy (10.6 eV for Mg) from the LSPRs (<1 to 6 eV for Mg) due to the confinement of the latter.^37^ This bulk plasmon absorption provides means to identify and map the presence of metallic Mg, independently from the LSPRs. STEM-EELS was used to map the Mg bulk plasmon at 10.6 eV and confirmed the presence of metallic Mg (Fig. 1b and S1†). Further, the formation of a polymer shell was visualized in STEM-EDS (Fig. 1c and S1†), where C and O signals prominently feature beyond the Mg signal. In addition to the broad ensemble plasmonic response of Mg suspension, which spans the UV-VIS-NIR (<300 to >1000 nm), the condensation of a PDA shell further comes with an intense absorption band in the UV-VIS around 460 nm that increases and shifts into the visible range with reaction time (Fig. S2†), eventually dominating the extinction spectrum and preventing us from observing the LSPR signature of coated structures optically.

**Fig. 2:**
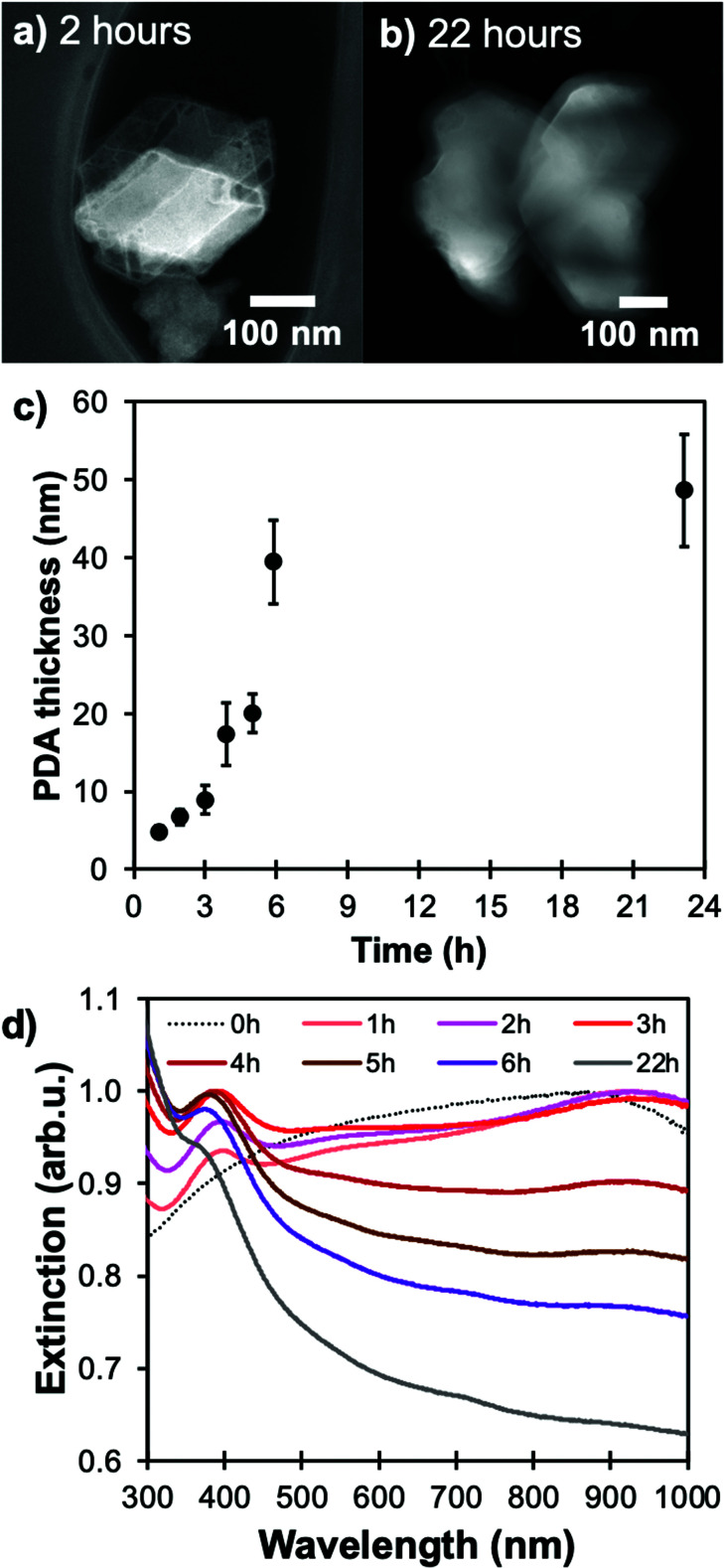
Shell thickness control for Mg@PDA. Representative STEM-HAADF images of Mg@PDA NPs for reaction times of (a) two hours and (b) 22 hours, and influence of the change in reaction time during the dopamine polymerization reaction as measured in (c) STEM-HAADF measurements of shell thicknesses (*N* > 50 for each reaction), and (d) in UV-VIS spectroscopy.

Over time, the PDA coating grows and stopping the reaction at specific times allows for thickness control. We obtained shells varying from 5 to over 50 nm for reaction times from one to 22 hours, respectively, as measured by STEM-HAADF (Fig. 2, S3 and Table S1†). The increasingly strong PDA absorption band between 300 and 460 nm shown in Fig. 2d also corresponds to an increase in PDA shell thickness over time.^38^

### Magnesium@silica core–shell particles

A modified Stöber condensation methodology inspired by Van Blaaderen *et al.*^9,23,39^ was used to prepare magnesium-core@silica-shell (Mg@SiO_2_) structures. We have compared the effect of adding the same molar quantity (0.150 mol L^−1^) of different bases of decreasing alkalinity; ammonium, DMA, and triethylamine (TEA), respectively. Since condensation of a silica shell did not happen with TEA and ammonium (Fig. S4†) led to the production of a silica shell along with significant secondary silica NPs, all reactions were performed with DMA as the base catalyst.

**Fig. 3:**
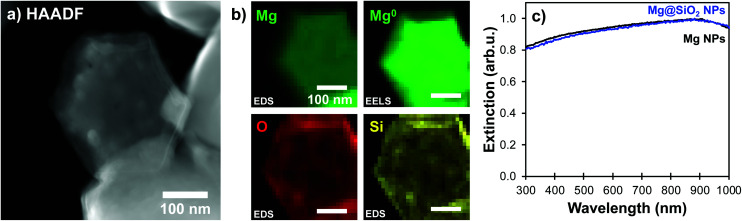
Mg@SiO_2_ with a 10 nm thick shell. (a) STEM-HAADF, (b) STEM-EDS maps for the K_α_ lines of Mg (1.25 keV), O (0.52 keV), and Si (1.74 keV), along with the STEM-EELS signature of the Mg bulk plasmon (10.6 eV), and (c) extinction spectra in UV-VIS spectrometry before (black) and after (blue) the Stöber condensation process. All scale bars, 100 nm.

The particles obtained were metallic Mg coated by SiO_2_. Using the Mg bulk plasmon signal attributed to Mg^0^, we confirmed that shapes are conserved, and metallic Mg remains in the core of the particle after silica coating (Fig. 3). Further, the coating consists of Si and O, which we confirmed using STEM-EDS maps. These, shown in Fig. 3b indeed reveal overlapping signals from the O and Si K_α_ lines around the well-formed Mg core (Fig. 3a, b, S5 and S6†). Further confirmation of the unchanged Mg NP structure and metallic character comes from the UV-VIS extinction spectra (Fig. 3c): the plasmonic response of Mg NPs remains unchanged after condensation of the silica layer, except for a slight red-shift due to the change in local refractive index around the Mg core.

**Fig. 4:**
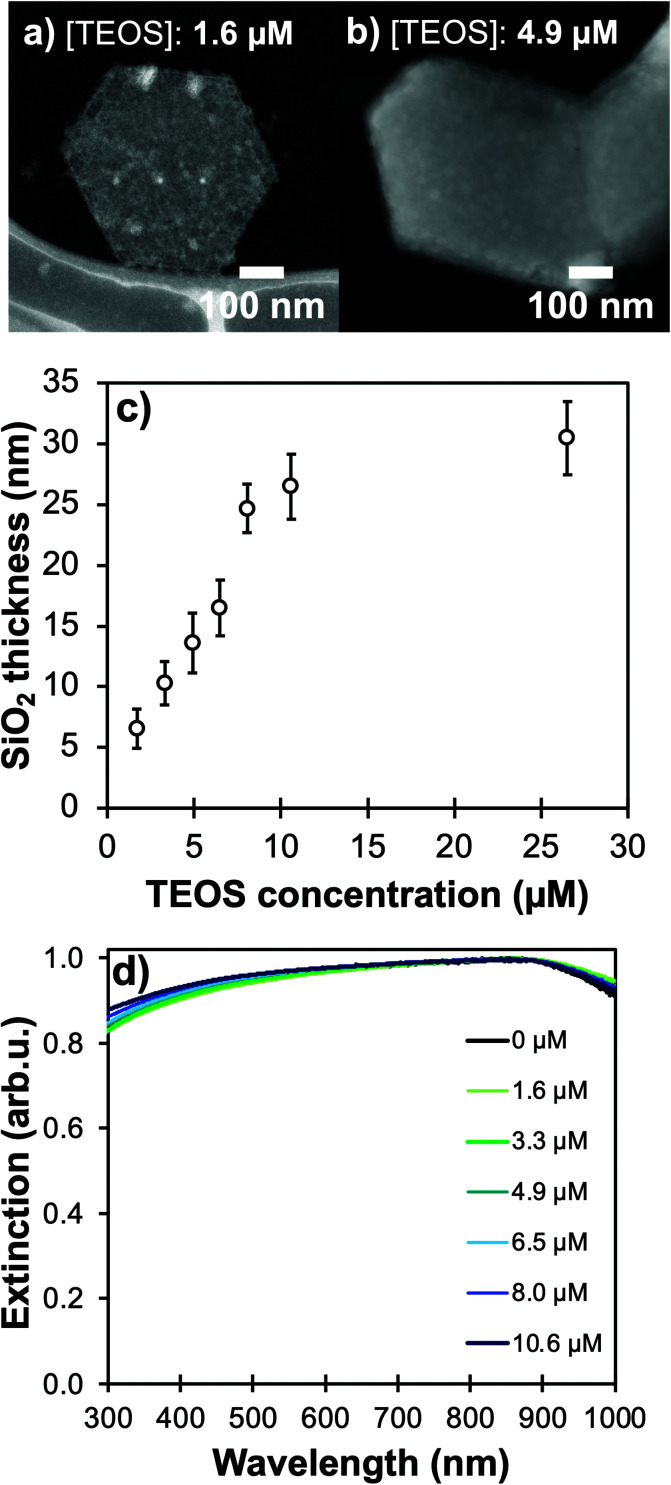
Controllable silica shells on Mg NPs. STEM-HAADF of NPs coated with TEOS concentration of (a) 1.6 μM with a shell of 6 ± 4 nm, and (b) 4.9 μM for a shell of 15 ± 2 nm. Effect of changes in TEOS concentration in the Stöber reaction medium as measured in (c) STEM-HAADF measurements of shell thicknesses (*N* > 50 for each reaction) and (d) UV-VIS spectroscopy.

Using higher concentrations of TEOS in the reaction medium improves the homogeneity of silica shells (Fig. 4a and b, Table S2†). The ease of silica condensation depends on the surface chemistry of the NPs; for instance, polyvinyl pyrrolidone-capped Au NPs and tannic acid-capped Ag NPs need extensive cleaning or ligand exchange steps before proceeding to a controlled Stöber reaction with uniform coating thickness and minimal secondary nucleation.^40^ For Mg NPs, the MgO layer and ligand-free core synthesis appear to facilitate the coating process, but the surface of the core–shell colloids remains rough even after reaction optimization (Fig. 3a, 4a and b), as reflected by the high standard deviation on the thickness measurements (Fig. S6 and S7, Table S2†); this effect could be due to a relatively low affinity of silicates towards the stable MgO surface. This feature could be especially interesting for biological applications as surface roughness has been demonstrated to be beneficial for non-invasive interaction with eukaryote cells and bacteria.^41^

Keeping the colloidal concentration constant, SiO_2_ shell thickness can be controlled by the concentration of TEOS in the reaction medium for Au- and Ag-based structures described in the literature.^23,24,42^ For Mg NPs, changing [TEOS] from 1.6 μM to 10 μM indeed leads to shell thicknesses smoothly increasing from 5 to 25 nm with good control and batch-to-batch reproducibility (Fig. 4 and S7, Table S2†). As the shell thickness increases, a shift of the plasmon response towards lower energies is observed, as expected since the electric field is confined in a larger volume of SiO_2_; this behaviour is akin to that of silica-coated Ag, Au, Cu, and In.^24,42–44^ The shift observed in Fig. 4d also confirms that the electric field around Mg reaches into the SiO_2_ shell and is not solely confined to the 10 nm thick MgO passivation layer. For all thicknesses of SiO_2_, we confirmed that the Mg core is not fully oxidized by observing the presence of the Mg bulk plasmon and the unchanged shape of the Mg NPs extinction band.

A [TEOS] above 6 μM leads to secondary nucleation of smaller SiO_2_ NPs even when using DMA (Fig. S8†). The size difference between Mg@SiO_2_ NPs and smaller SiO_2_ NPs enables efficient removal of the latter by additional centrifugation steps at a slower speed (3000 RCF), in the event thicker shells are desired.

### Stability of core–shell NPs in water

**Fig. 5:**
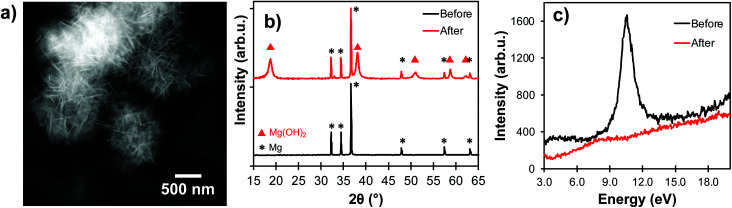
Bare Mg NPs degradation after exposure to water for 30 minutes. (a) STEM-HAADF, (b) X-ray diffraction, and (c) STEM-EELS, with a disappearance of the Mg bulk plasmon (10.6 eV).

While Mg NPs are stable for many weeks as a powder in air due to their thin native oxide layer,^3^ the Pourbaix diagram of Mg indicates that, at most pH and electrochemical potentials, the passivating oxidation layer forms a soluble species (Mg^2+^ or Mg(OH)_2_) when exposed to water, which reveals the vulnerable Mg^0^ core.^45^ Much like when Al NPs are exposed to aqueous solutions,^25^ the morphology of Mg NPs changes drastically after contact with water (Fig. 5a). This is due to a change in chemical composition from mostly metallic to hydrated MgO (Mg(OH)_2_), as confirmed by X-ray diffraction of unprotected Mg NPs exposed to water for 30 minutes (Fig. 5b). Further evidence of this oxidation is provided by the vanishing of the Mg bulk plasmon in STEM-EELS (Fig. 5c), and that of the optical plasmonic signature (Fig. S9†).

**Fig. 6:**
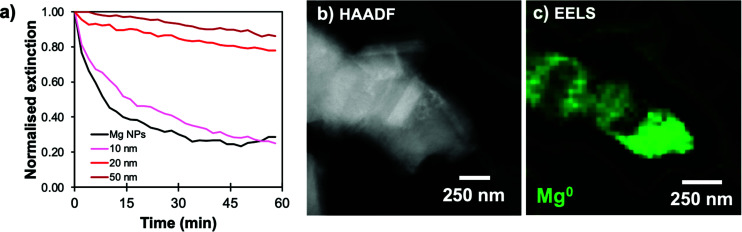
Stabilization of Mg@PDA NPs with shell thickness 20 nm. (a) Kinetics of degradation, viewed by extinction decrease in water at 900 nm, (b) STEM-HAADF of the sample after one hour in water, and (c) STEM-EELS signature of the Mg bulk plasmon (10.6 eV).

The shells synthesised here help protect Mg from oxidation in water. Since the oxidized forms of Mg are non-plasmonic, the dissolution/oxidation kinetics can be measured by following the decrease of extinction over time. The behaviour of Mg, Mg@SiO_2_ and Mg@PDA NPs of comparable shell thicknesses was studied in water, in 5 vol% water in isopropanol (IPA) solution, and in IPA (Fig. S10†). In all cases, PDA-based structures proved more stable than their silica counterparts (Fig. 6 and Fig. S11–S13†). In the case of the 5% solution of water in IPA, the extinction of all core–shell samples decreased for the first 15 minutes, but NPs retained their plasmonic properties after 30 minutes (Fig. S12†). However, in water, the behaviour of Mg@SiO_2_ NPs was barely distinguishable from that of bare Mg NPs, which lost most optical activity within 15 minutes. This can be attributed to the rough, non-uniform morphology of the silica shell that does not fully cover the MgO layer underneath (Fig. 4, S7 and S8†). PDA shells of a thickness >20 nm increased the time that Mg NPs were stable in water from 15 minutes to over 60 minutes. Moreover, while literature reports pore sizes smaller than 2 nm by N_2_ sorption for SiO_2_ prepared by Stöber-like sol–gel processes,^46^ similar investigations or comparisons with PDA-based nanostructures remains to be done but our results suggest smaller porosity.

**Fig. 7:**
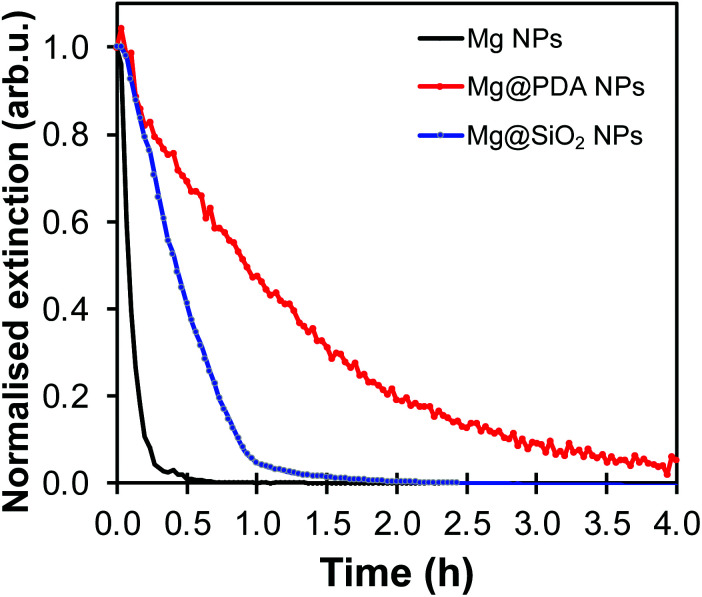
UV-VIS oxidation kinetics by the extinction decrease at 900 nm in water for Mg NPs (black), Mg@PDA NPs (20 nm thick shell, in red), and Mg@SiO_2_ NPs (20 nm thick shell, in blue).

The shell's thickness influences its stabilizing properties, where, as expected, a thicker PDA shell led to better protection against oxidation. For example, Mg@PDA NPs with a 20 nm thick shell (four-hour reaction time) were stable for well over an hour in aqueous suspension as shown by their retained optical properties (Fig. 6a). Stability increased marginally from 20 nm to 50 nm thick shells, however, a thin PDA shell is desirable to minimise interference from the PDA's own optical properties. Further, we confirmed that the core of the Mg@PDA particles exposed to water, cleaned by centrifugation, and redispersed in anhydrous IPA remains metallic with STEM-EELS (Fig. 6b, c and S14†). The size of the plasmonic core within the remaining passivation layer decreased significantly in water (Fig. S14†), but nevertheless remained metallic. While this plasmonic core represents a smaller proportion of the full NP volume, the improved stability of Mg@PDA core–shell NPs in water is indeed reproducible, as observed for a variety different syntheses and Mg NPs of different sizes (Fig. S15†). Indeed, we estimate that 50% of the optical response remains for Mg, Mg@SiO_2_ (20 nm thick), and Mg@PDA NPs (20 nm thick) after 5, 12, and 56 minutes in water, respectively, and it takes 15 minutes, 1 hour, and 3.5 hours before the same suspensions’ broad ensemble extinction signatures decrease by 95% (Fig. 7).

## Conclusion

In this work, we described the synthesis of a variety of core–shell structures based on Mg NPs and studied their stability in different aqueous environments. The Mg@PDA NPs obtained were uniform, and their shell thickness was modulated by studying and utilizing the reaction kinetics, producing PDA layers from 5 to >50 nm after reaction times from one to 22 hours. We demonstrated that Mg@PDA NPs are stable in aqueous solutions and retain the metallic character and plasmonic properties of Mg for several hours, as characterized by UV-VIS spectroscopy and STEM-EELS. The shell thickness of Mg@SiO_2_ NPs was controlled by varying the stoichiometry of TEOS in the reaction medium and by using DMA as a weak alkaline catalyst. Mg@SiO_2_ could be dispersed in 5% water/IPA but were not stable in fully aqueous conditions, with the plasmonic properties being lost within an hour.

The encapsulation of Mg NPs allows the expansion of Mg's plasmonic application by stabilizing this earth-abundant structure in aqueous solutions. This strategy should allow Mg to enter the field of applied plasmonics and compete with the standard Au and Ag systems. For instance, the addition of the well-known anchoring chemistry enabled by silica and polydopamine will allow the evaluation and comparison of Mg's performance in metal-enhanced fluorescence (MEF) and surface-enhanced Raman spectroscopy (SERS) of commercial dyes across the UV-VIS-NIR range. Further, improved stability of these colloids in water-containing or aqueous solutions unlocks application for sensing or therapy in short durations, capitalizing on Mg's biocompatibility.

## Materials and methods

### Chemicals and reagents

Anhydrous tetrahydrofuran (THF), anhydrous isopropanol (IPA), ethanol (EtOH), naphthalene, lithium pellets, di-*n*-butylmagnesium (1.0 M in heptane), tetraethyl orthosilicate (TEOS), ammonium hydroxide (28–30% in water), dimethylamine (DMA, 40% in water), triethylamine, dopamine hydrochloride, ethylenediaminetetraacetic acid (EDTA), and Eriochrome Black T were purchased from Sigma-Aldrich and used as supplied. Before use, all glassware was washed with aqua regia (1 : 3 HNO_3_ : HCl) and flame-dried under vacuum. (**Caution**: Aqua regia solutions are dangerous and should be used with extreme care; these solutions should never be stored in closed containers.)

### Air-free synthesis of Mg NPs

The air-free synthesis of Mg NPs used in this work is described in previous work from our group and others.^3,47^ Briefly, 2.12 g naphthalene, 0.112 g lithium, and 20 mL anhydrous THF were added to a Schlenk flask under argon atmosphere and sonicated for one hour, forming a deep green solution of lithium naphthalenide. 23 mL anhydrous THF and 7 mL di-*n*-butylmagnesium in heptane (1.0 M) were then added under argon atmosphere and left to stir for 16 hours with a magnetic stirring bar. The reaction was quenched by addition of 20 mL anhydrous IPA, and the product recovered by centrifugation and redispersion in anhydrous THF twice and anhydrous IPA twice to remove residual lithium, naphthalene, and organic by-products.

The Mg content of the resultant solution was estimated and diluted, if needed, to normalize the amount of colloidal nucleation points between different synthetic batches. To do so, the concentration of Mg NPs was estimated by complexometry titration experiments of dissolved Mg^2+^ ions with ethylenediaminetetraacetic acid (EDTA) and using Eriochrome Black T as indicator.^48,49^ An aliquot of 0.1 mL was extracted from the suspension, centrifuged at 8000 RCF for 10 minutes before removing the supernatant and dissolving the Mg NPs in 0.1 mL of 0.1 M nitric acid. This volume was added into a mixture of 30 mL DI water, 5 mL of an ammonium chloride/ammonia buffer (100 mM, pH 9), and 0.1 mL of the indicator solution (10 mM in ethanol with added hydroxylamine, 400 mM), before titration with a 1 mM EDTA solution until a stable blue colour (absorbance at 615 nm) was obtained.

### Preparation of Mg–polydopamine NPs (Mg@PDA)

Controlled condensation of polydopamine shells on Mg NPs was achieved by kinetic aliquots of dopamine polymerization in alkaline conditions (pH > 8.5). Briefly, 1.0 mL of as-prepared Mg NPs in IPA were added to 10 mL of a 4 mg mL^−1^ dopamine hydrochloride solution in ethanol, and 0.5 mL of DMA was added for reaction times between one and 22 hours (detailed in Table S1†). The mixture was purified by centrifuging twice at 8000 RCF for 10 minutes and NPs were dispersed in anhydrous ethanol.

### Preparation of Mg–silica NPs (Mg@SiO_2_)

Mg NPs were protected by a silica shell using a modified Stöber method with a weaker base (DMA instead of ammonium hydroxide).^9,23,24^ After synthesis, 0.6 mL of Mg NPs suspended in IPA were added to 4.0 mL EtOH before subsequent addition of 0.25 mL of a TEOS/EtOH solution of varying concentration (detailed in Table S2†). After thorough mixing, 0.10 mL of dimethylamine (40% in water) was added to the mixture and left to stir for 20–24 hours. Particles were then purified by centrifugation; firstly, at 8000 RCF for 15 minutes, and then five times at 3000 RCF for 5 minutes, before being redispersed in 0.4 mL EtOH.

### Characterisation

UV-VIS spectra were measured on an Evolution 220 spectrometer (ThermoFisher, UK) in quartz cuvettes under stirring at 25 °C. Analyses in 100% water conditions were achieved by centrifuging core–shell NPs at 8000 RCF before dispersing in water or IPA or a mixture thereof, and quickly starting the spectrometry characterisation. After the experiment, which typically lasted one hour, the NPs were pelleted down by two centrifugation steps at 8000 RCF for 10 minutes, followed by dispersion in anhydrous IPA before their characterisation in SEM (scanning electron microscopy) and STEM (scanning transmission electron microscopy).

Samples were drop cast on Si wafers for SEM imaging performed on a Quanta-650F Field Emission Gun Scanning Electron Microscope operated at 5 kV and equipped with an ETD detector for SE imaging. TEM and STEM analyses were performed on NPs drop cast on a Cu-supported lacey ultrathin carbon membranes. TEM, STEM, STEM-EELS for composition mapping, and STEM-EDS were acquired at 200 kV on a FEI Osiris STEM equipped with a Bruker Super-X quadruple EDS detector, and a Gatan Enfinium ER 977 electron spectrometer. STEM-EDS maps and line scans were obtained by integrating the K_α_ lines of Mg (1.25 keV), O (0.53 keV), Si (1.74 keV), and C (0.28 keV) after background subtraction.

XRD analyses were performed on a Bruker D8 DAVINCI with position sensitive detector (LynxEye EX) in coupled theta/2theta mode with a scan range of 15–65° and time per step of 0.68 seconds (0.01° per step). The source is Cu K_α_, and samples were drop cast onto silicon low-background holders.

## Conflicts of interest

The authors declare no conflict of interest.

## Supplementary Material

NR-013-D1NR06139A-s001
