# Supplementary material for: Improving the stability of plasmonic magnesium nanoparticles in aqueous media
Source: Nanoscale. 2021 Nov 29;13(48):20649–56. doi: 10.1039/d1nr06139a (PMC8675025; doi:10.1039/d1nr06139a)
Supplement: NR-013-D1NR06139A-s001 [file NR-013-D1NR06139A-s001.pdf]

Electronic Supplementary Material (ESI) for Nanoscale.
This journal is © The Royal Society of Chemistry 2021

Electronic Supplementary Information

Improving the stability of plasmonic magnesium nanoparticles in aqueous media

Jérémie Asselin[1,2], Elizabeth R. Hopper[1,2,3], Emilie Ringe[1,2]*

1. Department of Materials Science and Metallurgy, University of Cambridge, Cambridge, United Kingdom, CB3 0FS
2. Department of Earth Sciences, University of Cambridge, Downing Street, Cambridge, United Kingdom, CB2 3EQ
3. Department of Chemical Engineering and Biotechnology, University of Cambridge, Cambridge, United Kingdom, CB3 0AS

* Corresponding author: er407@cam.ac.uk; +44 (0)1223 334300 (ph.), +44 (0)1223 334567 (fax).

1. *Magnesium@polydopamine core-shell particles*

The synthesis of a polydopamine (PDA) shell on magnesium nanoparticles follows a methodology where the concentration of dopamine hydrochloride solution in ethanol is kept stable at 4 mg/mL and aliquots are taken at different times to tune the final PDA thickness. Solvent and solutions are mixed sequentially as follows:

1. 80 mg of dopamine hydrochloride
2. 20 mL ethanol
3. 2.0 mL of Mg NPs/isopropanol suspension (Mg concentration approx. 25 mmol/L)
4. 1.0 mL of dimethylamine (40% in water)

Syntheses can be scaled proportionally to prepare more or fewer core-shell NPs with reproducible result.

Table S1. Reaction time conditions and average measurements for the condensation of Mg@PDA NPs with different shell thicknesses.

Reaction time (hours)	PDA thickness (nm)	Secondary nucleation?
1	4.8 ± 0.8	No
2	6.7 ± 0.9	No
3	9 ± 2	No
4	17 ± 3	No
5	20 ± 2	No
6	39 ± 5	No
22	48 ± 7	No

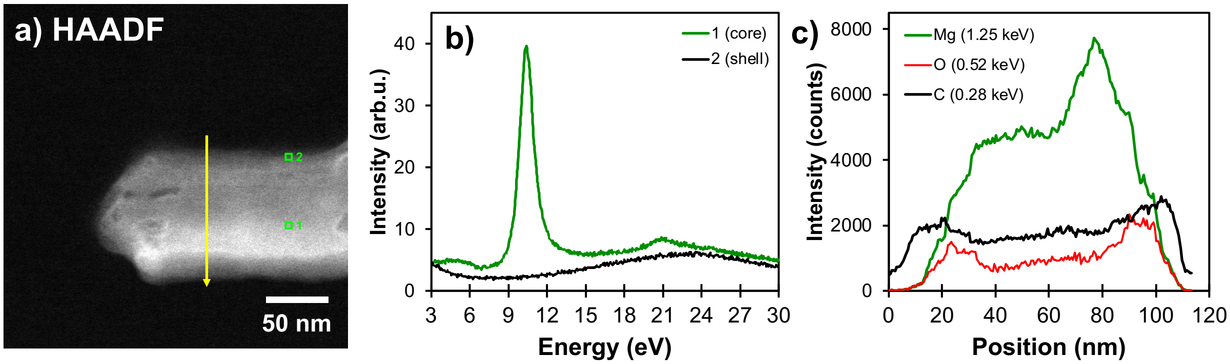

Figure S1. STEM characterisation of a single Mg@PDA NP with a) HAADF and the regions of interest presented in other panels, b) EELS showing the Mg bulk plasmon at 10.6 eV for the metallic core, and c) EDS line profiles for the K_α lines of Mg (1.25 keV), O (0.52 keV), and C (0.28 keV) along the line shown in a).

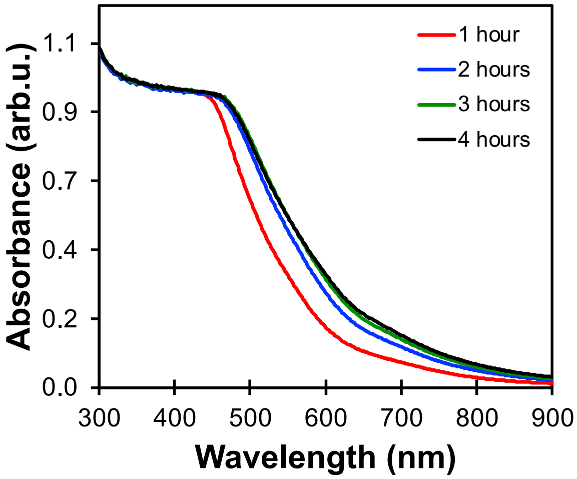

Figure S2. UV-VIS absorption of reaction supernatants from the media of Mg@PDA NPs between 1 to 4 hours.

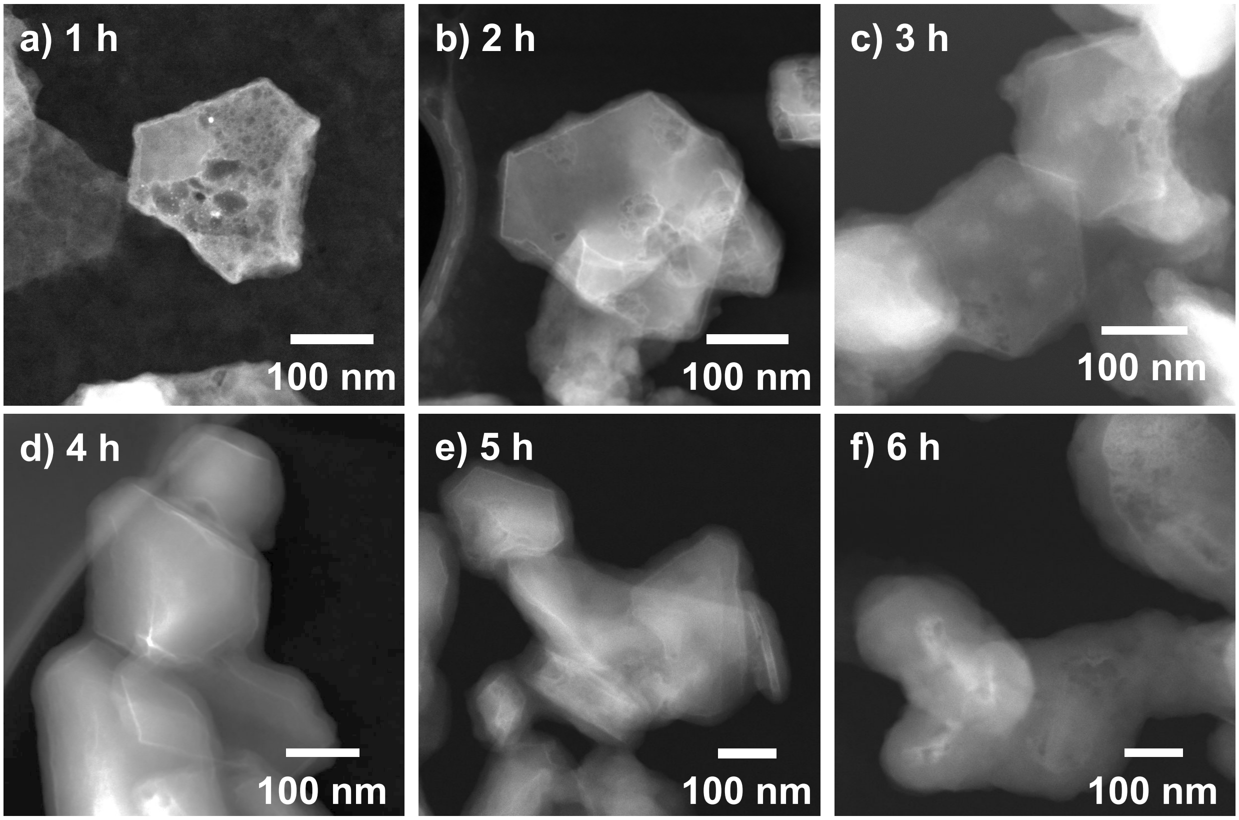

Figure S3. Representative STEM-HAADF micrographs of Mg@PDA NPs prepared from different reaction times ranging from 1 to 6 hours.

2. *Magnesium@silica core-shell particles*

The synthesis of a silica shell on magnesium nanoparticles follows a methodology where the concentration of the tetraethyl orthosilicate (TEOS) solution in ethanol is adjusted to tune the final silica thickness. Solvent and solutions are mixed sequentially as follows:

1. 4.0 mL ethanol
2. 0.25 mL of TEOS/ethanol solution (see Table S1)
3. 0.60 mL of Mg NPs/isopropanol suspension (Mg concentration approx. 25 mmol/L)
4. 0.10 mL of dimethylamine (40% in water)

Syntheses can be scaled proportionally to prepare more or fewer core-shell NPs with reproducible result.

Table S2. Experimental values for different silica shell thickness on Mg NPs for different TEOS solutions (N >50) and the effective TEOS concentration in the reaction medium. The last column indicates conditions where significant secondary nucleation of smaller SiO_2 NPs was observed.

TEOS solution (mL)	TEOS concentration (µmol/L)	SiO_2 thickness (nm)	Secondary nucleation?
30 µL in 5.0 mL	1.7	7 ± 2	No
60 µL in 5.0 mL	3.3	10 ± 2	No
75 µL in 5.0 mL	4.1	11 ± 2	No
90 µL in 5.0 mL	4.9	14 ± 3	Yes
120 µL in 5.0 mL	6.5	17 ± 2	Yes
150 µL in 5.0 mL	8.1	25 ± 2	Yes
200 µL in 5.0 mL	10.6	27 ± 3	Yes
500 µL in 5.0 mL	26.5	30 ± 4	Yes

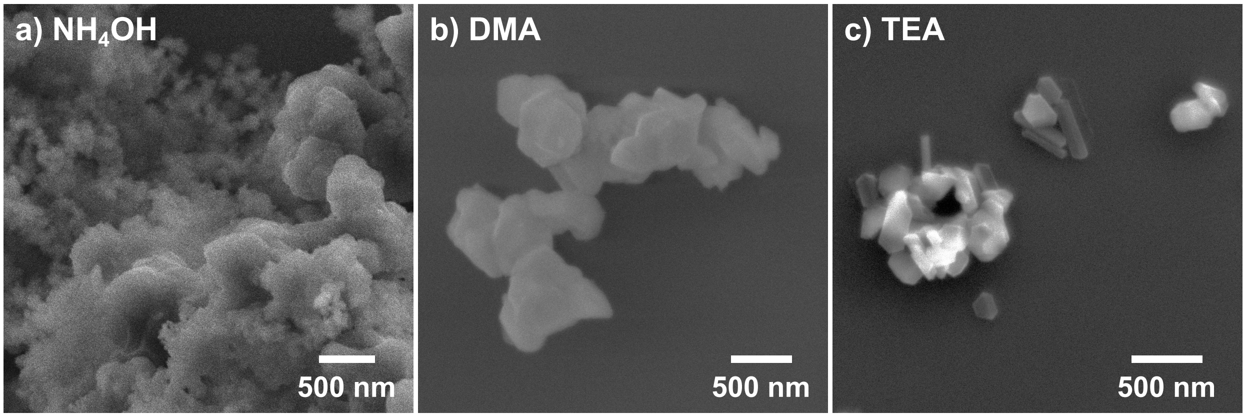

Figure S4. SEM micrographs of NPs after a modified Stöber reaction on Mg NPs with 3 μmol/L TEOS and 0.150 M of a) ammonium hydroxide, b) dimethylamine, and c) triethylamine as base catalyst. Particles with smoother edges and lighter contrast were determined to be covered with SiO_2.

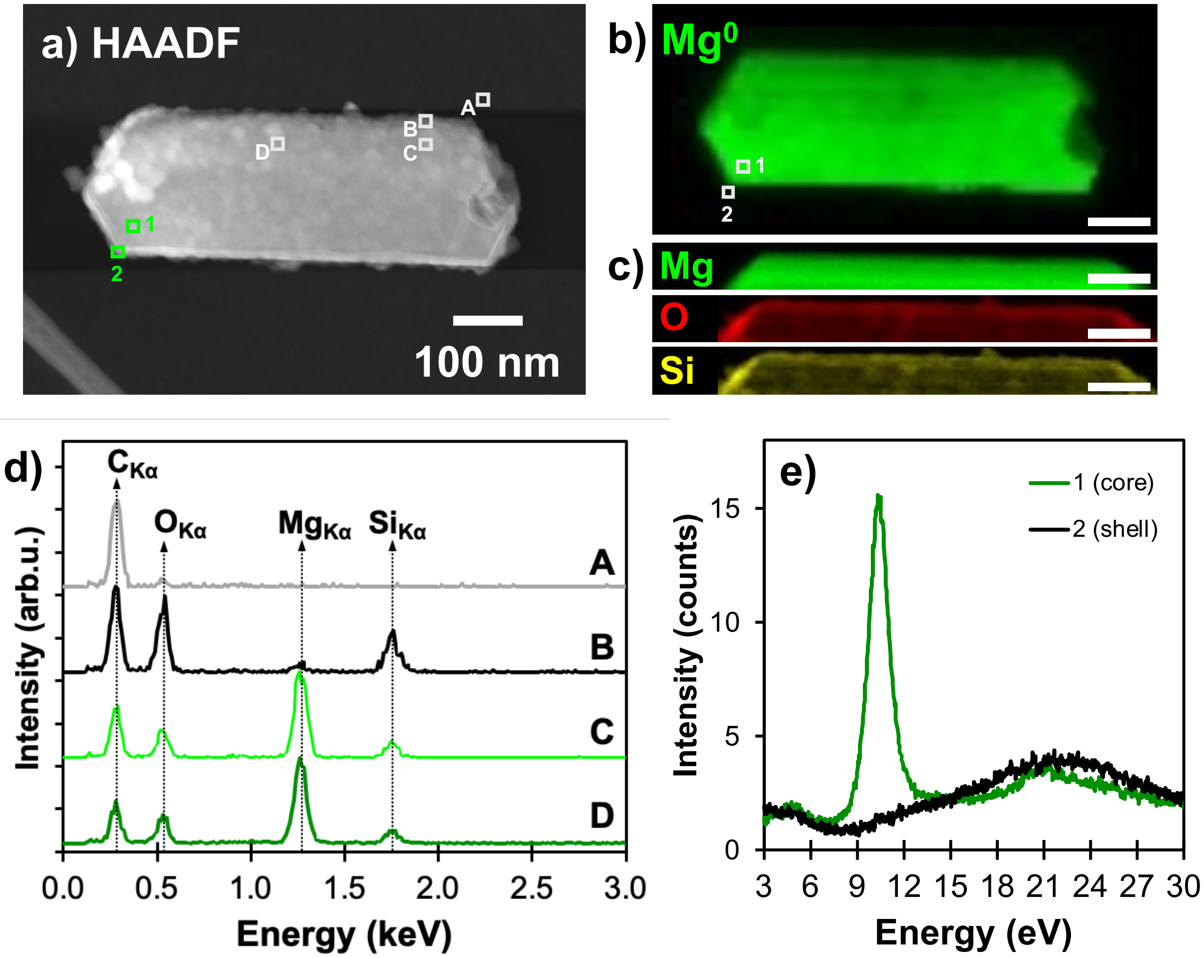

Figure S5. Characterisation of a single $Mg@SiO_2$ NP in a) STEM-HAADF along with the regions of interest presented in the other panels, b) STEM-EELS of the intensity for the Mg bulk plasmon at 10.6 eV, c) STEM-EDS for the K_α lines of Mg (1.25 keV), O (0.52 keV), and Si (1.74 keV). Single spectra acquired at specific spots are plotted in d) for STEM-EDS of the background (A), the shell (B), and the core (C and D), and in d) for STEM-EELS of the core (1) and the shell (2). All scale bars, 100 nm.

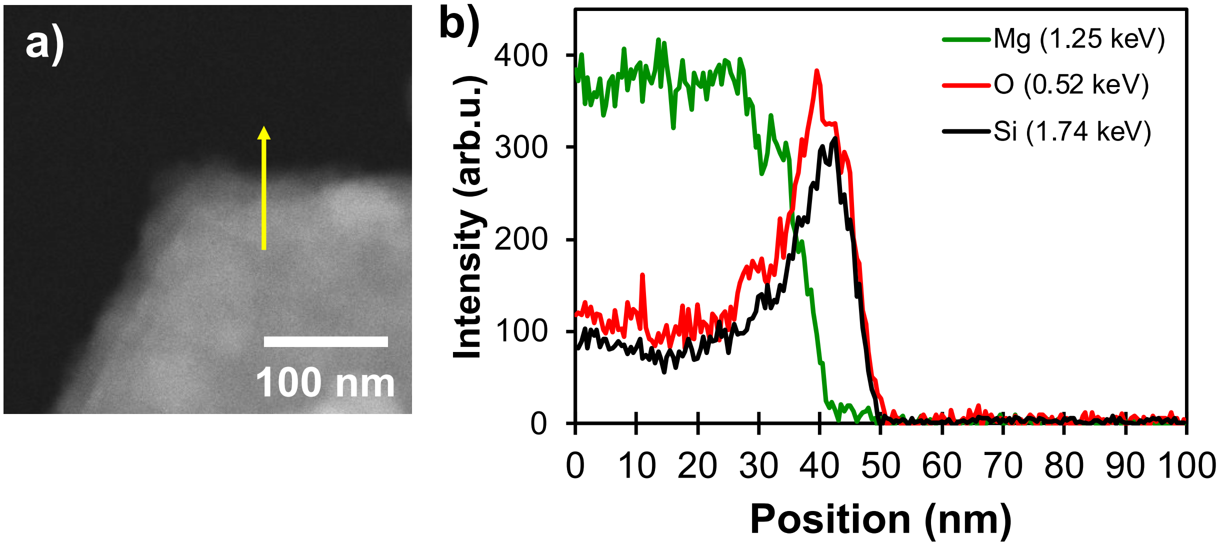

Figure S6. a) STEM-HAADF micrograph of the interface of a single Mg@SiO_2 NP and b) EDS line profiles of the K_α lines of Mg (1.25 keV), O (0.52 keV), and Si (1.74 keV) along the line shown in a).

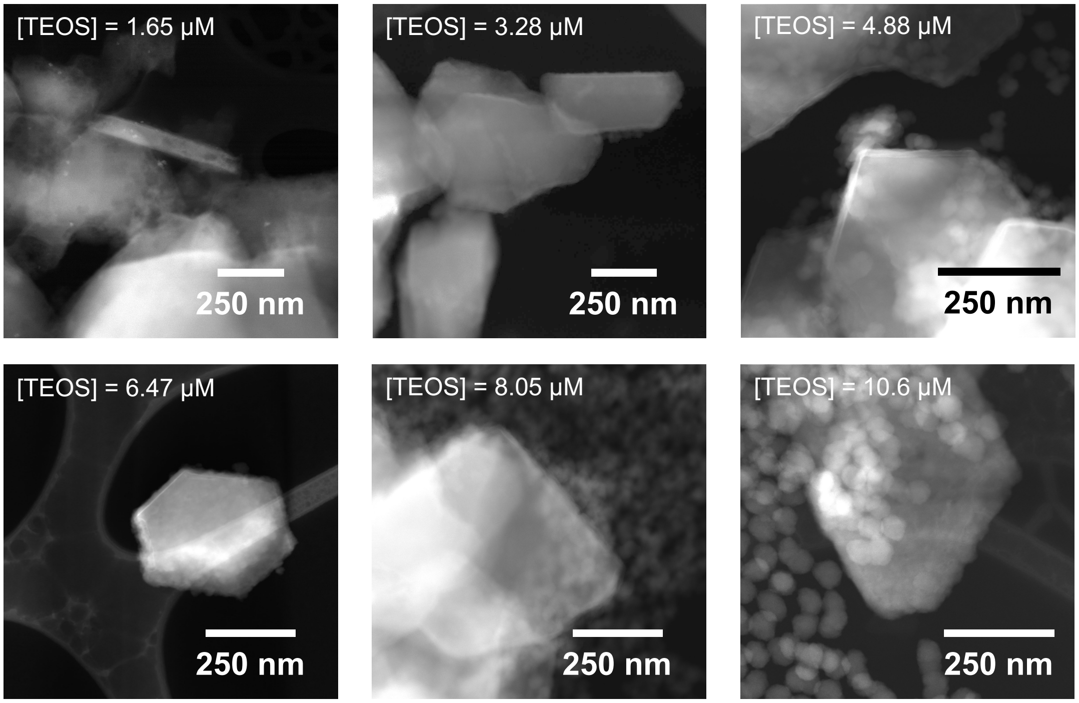

Figure S7. STEM-HAADF micrographs of Mg@SiO_2 NPs prepared with increasing TEOS concentration in the reaction medium, ranging from 1.65 to 10.6 µM.

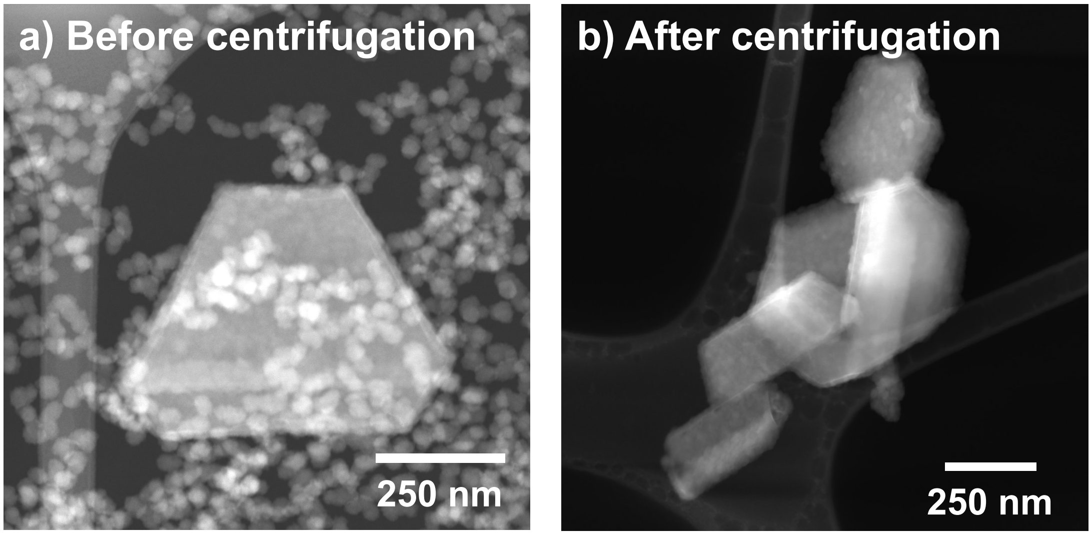

Figure S8. STEM-HAADF micrographs of core-shell Mg@SiO_2 NPs a) before and b) after further centrifugation steps at 3,000 RCF to separate them from secondary SiO_2 NPs and redispersion in isopropanol.

3. ***Stability of core-shell NPs in water***

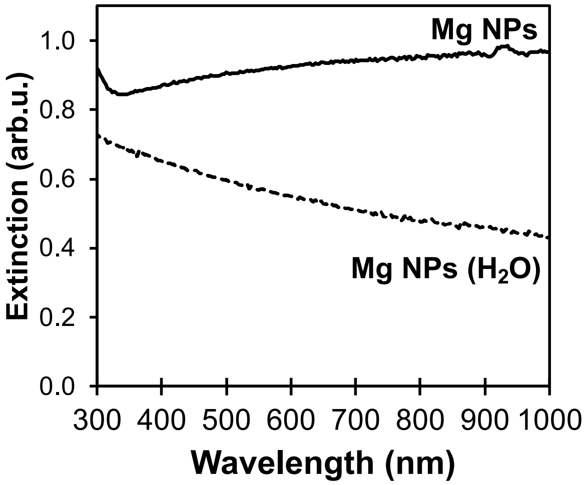

Figure S9. UV-VIS extinction from bare Mg NPs before (solid line) and after (dashed line) exposure to water for 1 hour.

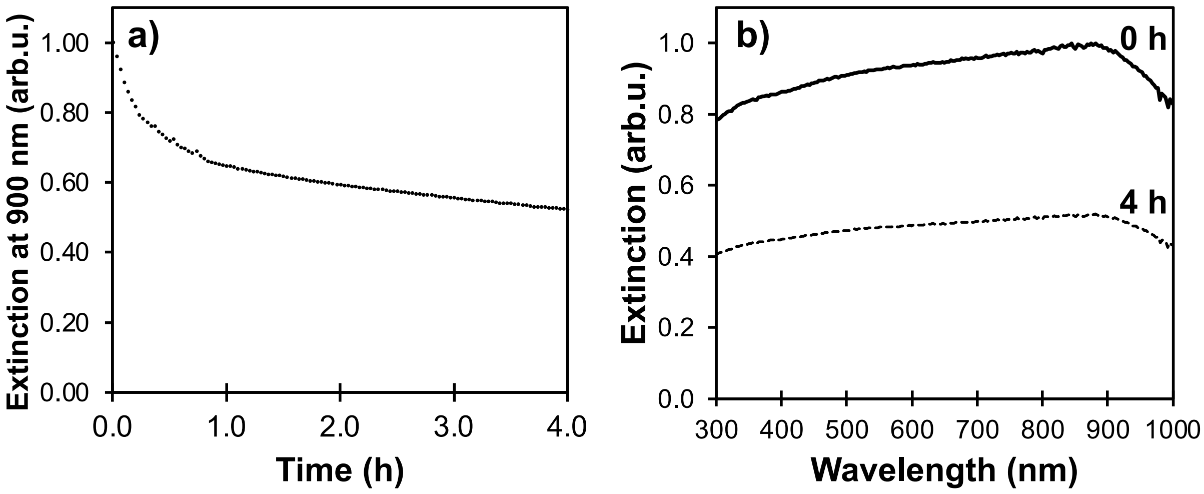

Figure S10. UV-VIS extinction of Mg NPs in IPA. a) Intensity decrease at 900 nm due to aggregation during measurements, and b) extinction spectra before (solid) and after (dashed) 4 hours in IPA. Upon further stirring and sonication, the sample's extinction returns back to its original intensity.

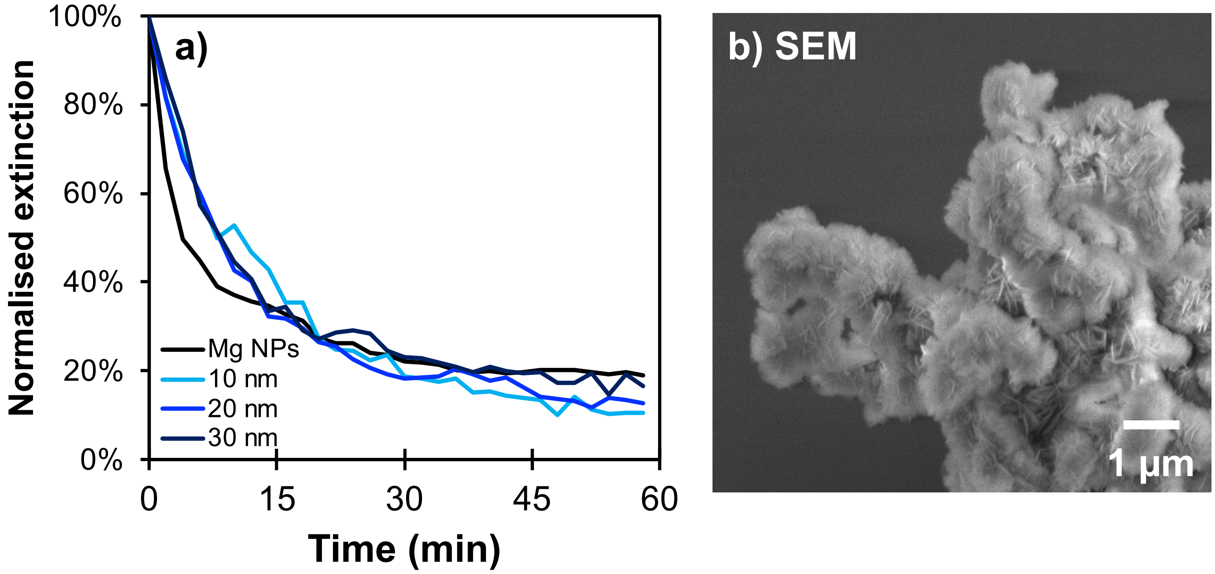

Figure S11. a) UV-VIS oxidation kinetics at 900 nm for bare Mg (black) and Mg@SiO_2 NPs with different shell thicknesses (shades of blue) in water for 1 hour, and b) SEM micrograph of the Mg@SiO_2 NPs (30 nm thick shell) after exposure to water.

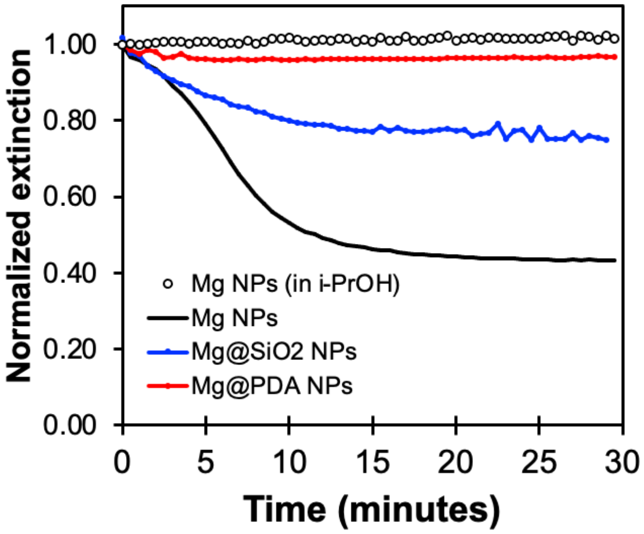

Figure S12. UV-VIS oxidation kinetics at 900 nm in 5%vol water-in-isopropanol (control: Mg suspension in isopropanol, white markers) for bare Mg NPs (black), Mg@SiO_2 NPs (blue) with a shell thickness of 17 nm (6.5 µM TEOS), and Mg@PDA NPs (red) with a shell thickness of 17 nm (4 hour reaction).

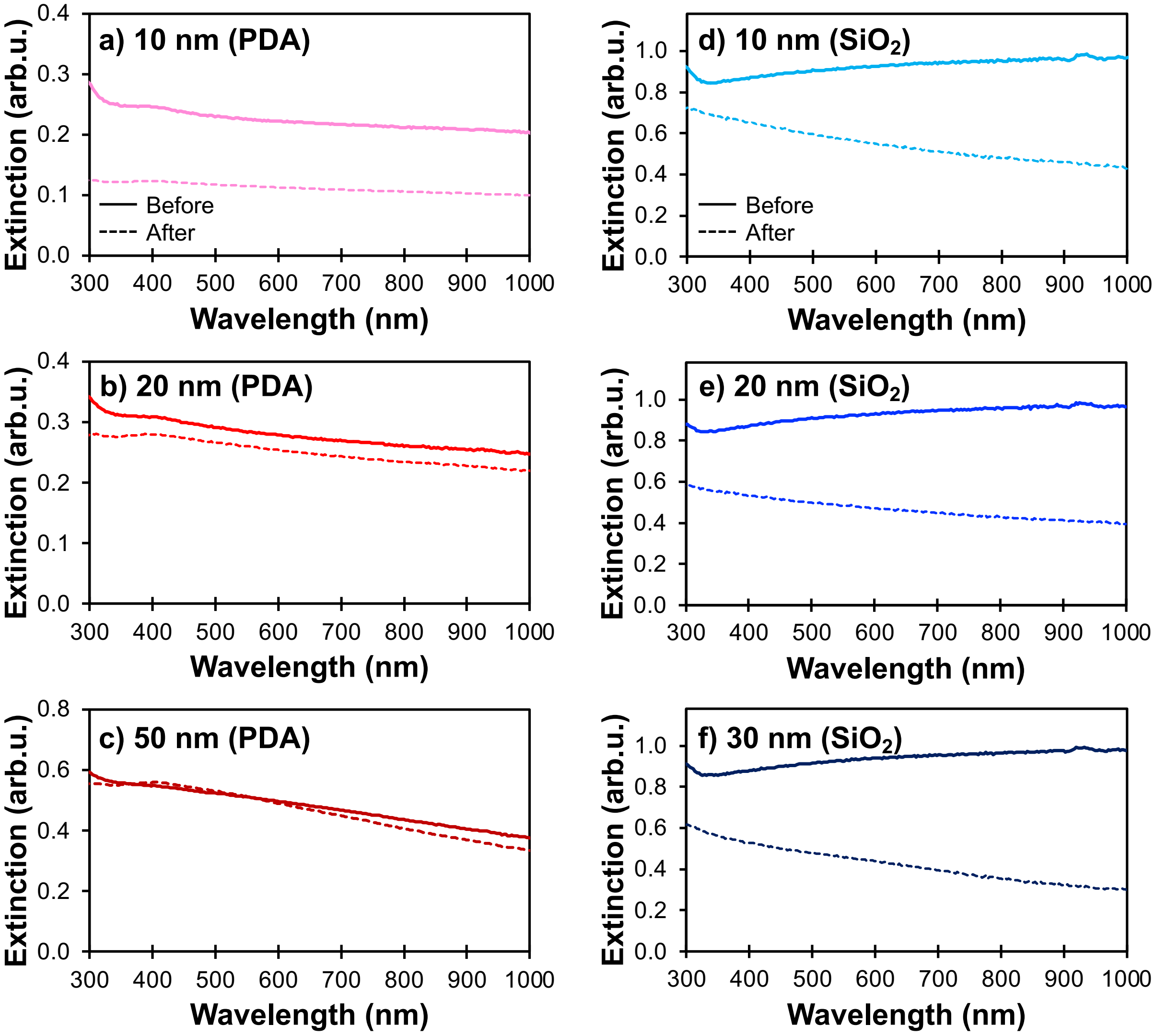

Figure S13. Changes in extinction in UV-VIS spectroscopy for Mg@PDA NPs with varying shell thicknesses a) 10 nm, b) 20 nm, c) 50 nm) and Mg@SiO_2 NPs d) 10 nm, e) 20 nm, f) 30 nm, where the signature before and after are represented by the solid and dashed lines, respectively.

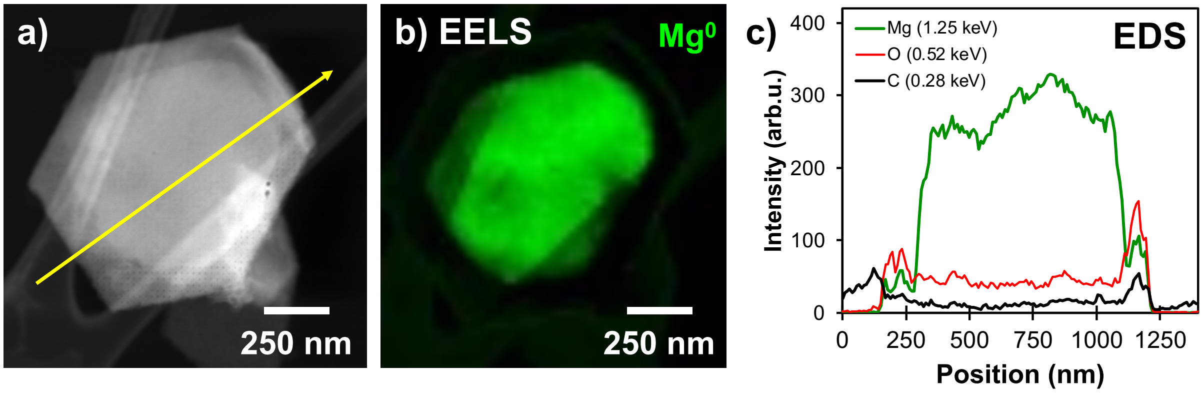

Figure S14. STEM characterisations of a single Mg@PDA NP (50 nm sample, 22 hour reaction time) after exposure to water for 1 hour in a) HAADF along with the region of interest for line profiles, b) EELS for the intensity of the Mg bulk plasmon at 10.6 eV, and c) EDS line profiles for the K_α lines of Mg (1.25 keV), O (0.52 keV), and C (0.28 keV) along the line shown in a).

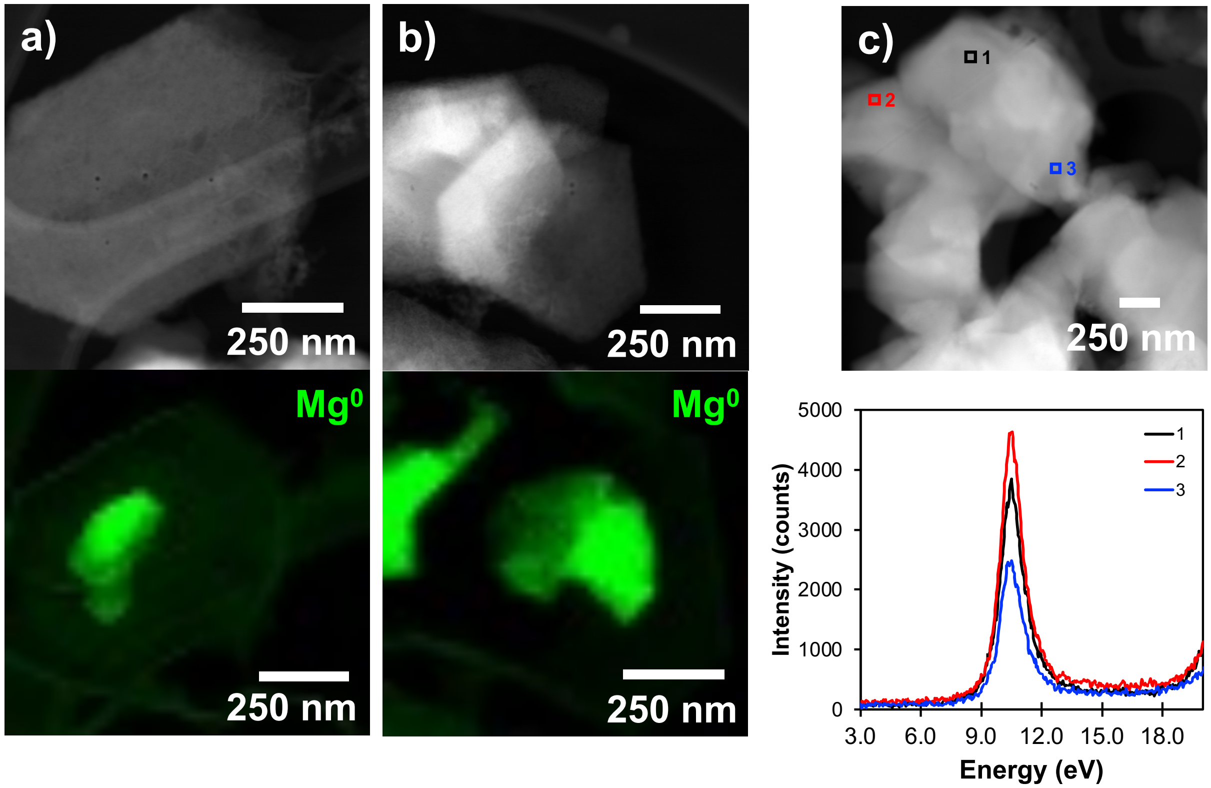

Figure S15. STEM characterisation of Mg@PDA NPs (20 nm sample, 4 hour reaction time) after exposure to DI water for 1 hour with HAADF and EELS maps (a and b), and multi-point EELS analyses (c).
